# 免疫抑制治疗下新冠病毒核酸反复复阳1例报告并文献复习

**DOI:** 10.3760/cma.j.issn.0253-2727.2023.06.016

**Published:** 2023-06

**Authors:** 倩 王, 琪然 张, 怡 张, 彤 陈

**Affiliations:** 1 复旦大学附属华山医院血液科，上海 200040 Department of Hematology, Huashan Hospital, Fudan University, Shanghai 200040, China; 2 复旦大学附属华山医院感染科，上海 200040 Department of Infectious Diseases, Huashan Hospital, Fudan University, Shanghai 200040, China

血液系统疾病患者因原发病及相关治疗常引起不同程度的免疫抑制。新型冠状病毒肺炎（coronavirus disease-2019，COVID-19）流行期间，部分免疫抑制状态的患者受到感染。免疫抑制患者感染新冠病毒后，一部分患者易发生重症新冠肺炎，另一部分患者尽管没有严重的症状，却不能清除体内病毒，而发生慢性感染，出现新冠病毒核酸持续或反复阳性的情况。现报告我院近期诊治的1例免疫抑制治疗下新冠病毒核酸反复复阳的患者并对相关文献进行复习，以提高对免疫低下患者新冠病毒持续感染的认识，以及探讨对需继续接受化疗或免疫抑制治疗的既往新冠病毒感染患者的管理措施。

## 病例资料

患者，女，59岁，因“反复发热3个月余”于2022年6月3日入血液科。患者2022年3月初开始无明显诱因下出现发热，体温最高39.1°C，无明显畏寒、寒战、咳嗽、咳痰、腹泻、腹痛、头痛症状，抗感染治疗疗效不佳，外院B超检查发现双侧腋下、耳后、颈部左侧淋巴结肿大。行颈部左侧淋巴结穿刺，病理示淋巴结结构消失，在T淋巴细胞增生活跃的背景中可见散在灶性分布核中等/大的B淋巴细胞浸润，淋巴瘤不能除外（由于穿刺组织过少，未行酶标检查）。2022年4月24日患者因新冠病毒核酸检测阳性隔离医学观察，4月27日转入新冠定点医院，住院期间有间歇性发热，体温最高38.7 °C，未吸氧状态下SpO_2_ 97％，血常规：WBC 3.13×10^9^/L，中性粒细胞绝对计数（ANC）2.64×10^9^/L，淋巴细胞绝对计数（ALC）0.22×10^9^/L，HGB 90 g/L，PLT 83×10^9^/L，降钙素原（PCT）10.90 µg/L，C反应蛋白（CRP）227.90 mg/L。胸部CT平扫未见明显感染征象。予哌拉西林-他唑巴坦 4.5 g每8 h 1次抗感染治疗后体温恢复正常。4月30日、5月1日患者2次复查新冠病毒核酸ORF1ab基因Ct值均>40，5月3日解除隔离转入我院感染科住院。5月4日查血常规：WBC 0.97×10^9^/L，HGB 70 g/L，PLT 63×10^9^/L。EBV-DNA（血浆）1.19×10^5^拷贝数/ml，CRP 81.56 mg/L，铁蛋白6 685 µg/L，IL-2R 6.144 U/L，纤维蛋白原（FIB）1.1 g/L，甘油三酯（TG）3.70 mmol/L，乳酸脱氢酶（LDH）699 U/L。自身免疫指标、肿瘤标志物均正常，HIV阴性。骨髓细胞学检查发现单核组织巨噬细胞比例增多伴活化，可见噬血细胞及少量不典型淋巴细胞，提示噬血性淋巴单核组织巨噬细胞增多可能。B超检查发现颈部双侧、双侧腋下、腹股沟多发肿大淋巴结，后腹膜髂血管旁可见散在肿大淋巴结，脾脏增大（14.0 cm×5.2 cm）。患者既往因罹患寻常性银屑病、关节病型银屑病30余年，使用阿达木单抗40 mg每2周1次治疗近10年。诊断为：①发热待查：EBV感染，淋巴瘤待排；②银屑病。5月4日予地塞米松15 mg/d联合静脉注射免疫球蛋白0.4 g·kg^−1^·d^−1^×5 d治疗，5月7日加用环孢素A（CsA）150 mg每日2次口服治疗。患者体温持续正常，5月13日复查血常规：WBC 3.33×10^9^/L，ANC 2.60×10^9^/L，ALC 0.62×10^9^/L，HGB 93 g/L，PLT 80×10^9^/L。CRP 3.14 mg/L，铁蛋白3 160 µg/L，IL-2R 2.199 U/L，FIB 2.0 g/L，LDH 342 U/L。5月13−15日患者连续3 d新冠病毒核酸检测阳性，同时环境采样均为阴性。5月15日患者再次转入定点医院住院，5月16日起地塞米松减量至10 mg/d，5月17日起予奈玛特韦/利托那韦（Paxlovid）300 mg每12 h 1次治疗5 d，考虑药物相互作用，同日CsA减量至75 mg每日2次，患者体温持续正常，监测血常规及CRP、铁蛋白、IL-2R、FIB、TG、LDH指标进一步改善，疗效评估维持部分缓解（PR）。胸部CT未见病毒性肺炎表现。定点医院住院期间连续监测新冠病毒核酸ORF1ab基因Ct值从22.50逐日升高，至5月24日患者新冠病毒核酸检测ORF1ab基因Ct值>40再次转入我院感染科住院，继续予地塞米松10 mg/d联合CsA 100 mg每日2次治疗，5月27日患者再次出现发热，体温高峰达39 °C，复查EBV-DNA（血浆）5.48×10^2^拷贝数/ml，EBV-DNA（全血）6.29×10^3^拷贝数/ml，血常规：WBC 3.21×10^9^/L，ANC 2.90×10^9^/L，ALC 0.21×10^9^/L，HGB 78 g/L，PLT 87×10^9^/L。CRP 65.16 mg/L，铁蛋白3 155 µg/L，IL-2R 3.311 U/L。外周血淋巴细胞亚群：CD3^+^ 87.27％，CD4^+^ 21.58％，CD8^+^ 61.91％，CD4/CD8 0.35，CD19^+^ 1.38％，CD20^+^ 1.10％。复查B超仍可见颈部双侧、锁骨上、腋下多发肿大淋巴结，脾脏增大。6月7日转入我院血液科予依托泊苷（100 mg/d，第1、4天）+环磷酰胺（1.1 g，第2天）+长春地辛（4 mg，第2天），联合地塞米松10 mg/d+CsA 100 mg每日2次+静脉注射免疫球蛋白0.4 g·kg^−1^·d^−1^×5 d治疗，患者体温再次恢复正常，6月15日患者血常规：WBC 0.12×10^9^/L，HGB 67 g/L，PLT 30×10^9^/L。骨髓细胞学检查提示骨髓抑制。EBV-DNA（血浆）低于监测下限，EBV-DNA（全血）1.39×10^3^拷贝数/ml，CRP 4.65 mg/L，铁蛋白1 049 µg/L，IL-2R 0.956 U/L。6月18日、6月19日患者新冠病毒核酸检测再次阳性（ORF1ab基因Ct值分别为31、26），同时新冠病毒抗原检测为阳性，环境及密接新冠病毒核酸检测均为阴性。患者肺部CT未见病毒性肺炎表现。6月16日电化学发光法检测患者血清新冠病毒中和抗体滴度为1∶16，抑制率44.20％。此后患者转入新冠定点医院治疗。

## 讨论及文献复习

本文描述了1例免疫抑制剂治疗患者确诊新冠病毒感染后，在2个月内多次出现口咽及鼻咽拭子新冠病毒核酸复阳的病例。我们全程描述患者每次新冠病毒复阳前免疫抑制治疗的方案及时长，希望通过本例报道，有助于提高对免疫低下患者新冠病毒持续感染的认识，并为既往新冠病毒阳性患者化疗或免疫抑制治疗的方案选择提供参考。

本例患者因银屑病长期使用阿达木单抗治疗，2022年3月出现反复发热伴多发淋巴结肿大，淋巴结活检病理见淋巴结结构消失以及散在灶性分布的核中等/大的B淋巴细胞浸润，但无法确诊或排除淋巴瘤，并伴有EBV活动。2022年4月患者出现鼻咽拭子RT-PCR检测新冠病毒核酸阳性，肺部CT阴性，确诊轻型新冠病毒肺炎。以患者首次新冠病毒核酸阳性为第0天，则其分别于第19天、第55天在两次强化免疫抑制治疗后（第1次：第10天开始使用地塞米松+CsA；第二次第44天开始依托泊苷+环磷酰胺+长春地辛+地塞米松+CsA）出现鼻咽拭子新冠病毒核酸复阳。患者首次确诊新冠病毒感染转阴后及后续两次新冠病毒核酸复阳期间，均每日接受新冠病毒核酸检测，结果均为阴性。后续我们为患者检测中和抗体（电化学发光法），其新冠中和抗体滴度仅1∶16，抑制率为44.20％。

新冠病毒感染转阴后复阳并不少见，既往的文献已有很多报道。新冠病毒核酸连续阴性后出院COVID-19患者中再阳性患者的比例从2.4％到69.2％不等，出院后持续1～38 d均有复阳的报道[1]。复阳原因包括前次出院时假阴性、复阳时样本的假阳性、再次感染、患者携带的无活性新冠病毒核酸、病毒再激活等。本例患者发生在国内，我国对新冠疫情防控有着较为严格的措施，该例患者从首次感染后核酸连续阴性出院，到后续两次复发期间，由于一直在病房接受噬血细胞综合征及原发病的诊治，患者及其陪护人员、同病房患者均遵守每日一次的核酸检测；阳性样本均经过复核检验和再次采样复检验证，因此出院时样本“假阴性”和复阳时样本“假阳性”的可能性基本排除。尽管本例患者未对阳性样本中的病毒核酸进行测序检测其是否为同一毒株，同样，由于患者及其接触的人员在其复阳前连续核酸检测的阴性、患者复阳时周围环境采样检测新冠核酸的阴性，均提示再次发生新冠病毒感染的可能性较小。

本例患者发生的情况可能性较大的原因是病毒持续携带，另一种是病毒的再激活。前者是既往报道中最常见的情况，Wölfel等[2]和Bullard等[3]研究显示，距患者出现症状起超过8 d时，即使能检测到较高载量的新冠病毒核酸，患者样本中也无法分离或培养出具有感染能力的病毒。van Kampen等[4]对129例新冠病毒感染患者不同时间点的690份呼吸道样本进行分析，进一步发现能分离出病毒的中位时间点为出现症状后8 d，超过出现症状后15.2 d时，只有不到5％的患者样本中能分离出有感染能力的病毒。Kang等[5]在285例COVID-19康复后核酸复阳的韩国患者样本中未发现活性病毒。Lu等[6]在中国广东省87例复阳患者的36份样本（14份鼻咽拭子、3份咽喉拭子和19份肛门拭子）接种到Vero细胞进行培养，但均无法培养出活病毒，并且全部测序失败。这些研究都提示大多数新冠病毒核酸检测复阳很可能检测到的是无活性的病毒RNA，而不是再激活。

但是，本例患者有免疫低下的基础，并且每次复阳均发生在新一疗程的免疫抑制治疗时，尤其是在第二次复阳时，患者鼻咽拭子新冠病毒核酸检测阳性发生在粒细胞缺乏事件后，并且我们在其取样复核过程中观察到了样本核酸Ct值迅速下降，且患者鼻咽拭子新冠抗原检测弱阳性，这一系列现象都提示本例患者不能排除病毒持续存在并且在强化免疫抑制治疗发生再激活和复制的可能性。而近期的一些研究也表明，健康人群在感染新冠病毒后，常常能够彻底清除病毒并建立持久的免疫；然而，免疫抑制患者感染新冠病毒后，一部分患者易发生重症新冠肺炎，而另一部分患者尽管没有严重的症状，却不能清除体内病毒，而发生持续慢性的感染，出现新冠核酸持续或反复阳性伴临床症状复发的情况。近期的文献中，对这类患者已有多例报道和描述。例如，Cabañero-Navalon等[7]报道了1例22岁患有常见变异型免疫缺陷病（common variable immunodeficiency，CVID），0天确诊COVID-19，居家隔离过程中，在第20、38、55、72天反复因为发热、低氧血症和肺部影像学进展入院诊治，并确认每次都在一些样本（鼻咽拭子、痰、肺泡灌洗液或血）中被证实有新冠病毒核酸阳性。Palomba等[8]报道了1例正在免疫球蛋白替代治疗的X 连锁无丙种球蛋白血症，其在0天确诊COVID-19住院治疗至肺炎缓解、鼻咽拭子新冠核酸阴性后出院，后再次因高热、腹泻入院，并且入院后反复在样本中检测到核酸阳性。Maponga等[9]报道了1例进展期HIV感染的患者，其在新冠病毒感染后十余天、1个月、2个月的检查中新冠病毒核酸持续阳性且载量较高，后在感染第3个月免疫重建后检测到核酸转阴。Sepulcri等[10]报道1例利妥昔单抗、苯达莫司汀、阿糖胞苷治疗中的套细胞淋巴瘤患者，鼻咽拭子样本上的新冠病毒RT-PCR 保持阳性 268 d。这些研究中，有的不仅在患者样本中通过PCR检测到核酸阳性，还通过测序和系统发育分析证实了复阳的样本病毒来源于同一株病毒的同一进化枝；有的还从复阳的样本中分离出了病毒[7],[9]。另外，这些病例报道中既往感染过的患者都没有产生有效的体液免疫。这些研究都提示免疫抑制的患者中，新冠病毒感染后有可能持续存在，不断复制，并且可能反复出现病毒的再激活和临床症状的复发。持续存在新冠病毒的藏匿位置可能为下呼吸道的上皮细胞，由于新冠病毒进入细胞的必需受体ACE2在下呼吸道中的表达远高于上呼吸道，病毒可以从上呼吸道迁移到下呼吸道并在后者中持续复制[11]。这可能也解释了鼻咽拭子检测结果可间断为阴性的原因，也与部分病例报道中，痰或肺泡灌洗液新冠核酸阳性但鼻咽拭子新冠核酸阴性的现象一致[7]。从理论上讲，如果患者经历的是病毒再激活，并且病毒载量较高、患者自身无法产生新冠中和抗体，其有可能成为传染源。然而，目前既缺乏对免疫抑制患者中再激活病毒传染性的探究，亦尚无与再阳性患者接触的人中的感染病例报道。

本例患者尽管经历过新冠病毒感染和复阳，其中和抗体滴度仍低下（1∶16），不足以提供对新冠病毒的保护作用，这与既往的报道一致。对于这类患者，部分研究报道抗病毒药物瑞德西韦可缓解症状，并与一些患者的病毒清除和持续改善有关，但在另一些患者中，似乎只会产生短暂的病毒载量下降，一旦治疗停止就会复发。更新的药物（如本例患者使用的Paxlovid或Molnupiravir等）尚无在新冠病毒持续感染中应用价值的报道。而部分研究证实使用恢复期血浆或合成单克隆抗体可以使病毒持续存在数月的免疫低下患者实现治疗后症状完全消失和病毒持续清除[8],[12]。

我们展示本例患者的临床特征和病程，希望能让大家关注既往感染新冠病毒的免疫抑制患者反复核酸复阳的可能性，尤其是在周期性化疗的患者中。在治疗方案方面，在能够治疗原发病的前提下，考虑尽量采用对患者免疫功能抑制相对较小的方案。另外，对于这类患者，我们希望有更多、更大样本的队列研究来回答我们：是否存在简单易行的方法区分其核酸复阳是无活性病毒核酸的排出，还是病毒的再活动？如果确实存在病毒再激活，这样的病例是否具有传染性？哪些化疗方案会更加容易引起病毒的再活动？这些问题的探究，会为我们管理和治疗这类人群提供宝贵的指导。
